# Supplementary material for: Informing children of their parent's illness: A systematic review of intervention programs with child outcomes in all health care settings globally from inception to 2019
Source: PLoS One. 2020 May 26;15(5):e0233696. doi: 10.1371/journal.pone.0233696 (PMC7250450; doi:10.1371/journal.pone.0233696)
Supplement: S1 Appendix — (DOCX) [file pone.0233696.s002.docx]

Appendix 1

Documentation of search strategies

University Library search consultation group

Date: January 2018

Topic/research question: Doctors talking to children about their parents’ disease

Name of researcher(s): Solvig Ekblad & Charlotte Oja, LIME

Librarian(s): Sabina Gillsund & Magdalena Svanberg

Databases:

1. Medline/PubMed (Ovid)
2. Web of Science Core Collection
3. PsycInfo (Ovid)
4. Cinahl
5. SveMed+

Total number of hits:

- Before deduplication: 12,201
- After deduplication: 8,011

Comments:

1. Medline/PubMed

| Interface: Ovid  Date of Search: 9 January 2018  Number of hits: 3,810  Comment: In Ovid, two or more words are automatically searched as phrases; i.e. no quotation marks are needed | Field labels   - exp/ = exploded MeSH term - / = non exploded MeSH term - .ti,ab,kf. = title, abstract and author keywords - adjx = within x words, regardless of order - * = truncation of word for alternate endings |
| --- | --- |
| 1. Child of Impaired Parents/  2. ((parent* or maternal* or paternal* or mother* or father* or famil*) adj4 (disorder* or illness* or ill or impairment or impaired or sick* or diagnos* or disease* or injur*)).ti,ab,kf.  3. (child* or adolescen* or teen* or offspring or daughter* or son or sons).ti,ab,kf.  4. 2 and 3  5. 1 or 4  6. Professional-Family Relations/  7. Counseling/  8. Social support/  9. Communication/  10. Disclosure/  11. Truth disclosure/  12. ((disclos* or intervent* or support* or communicat* or counsel* or encounter* or telling or talking or conversation*) adj3 (adolescen* or famil* or child* or teen*)).ti,ab,kf.  13. or/6-12  14. 5 and 13  15. remove duplicates from 14 | |

2. Web of Science Core Collection

| Interface: Clarivate Analytics  Date of Search: 9 January 2018  Number of hits: 2,216 | Field labels   - TS/Topic = title, abstract, author keywords and Keywords Plus - NEAR/x = within x words, regardless of order - * = truncation of word for alternate endings |
| --- | --- |
| #1 TS=((parent* OR maternal* OR paternal* OR mother* OR father* OR famil*) NEAR/3 (disorder* OR illness* OR ill OR impairment OR impaired OR sick* OR diagnos* OR disease* OR injur*))  #2 TS=(child* OR adolescen* OR teen* OR offspring OR daughter* OR son OR sons)  #3 TS=((disclos* OR intervent* OR support* OR communicat* OR counsel* OR encounter* OR telling OR talking OR conversation*) NEAR/2 (adolescen* OR famil* OR child* OR teen*))  #4 #1 AND #2 AND #3 | |

3. Psycinfo

| Interface: Ovid  Date of Search: 9 January 2018  Number of hits: 4,028  Comment: In Ovid, two or more words are automatically searched as phrases; i.e. no quotation marks are needed | Field labels   - exp/ = exploded controlled term - / = non exploded controlled term - .ti,ab,id. = title, abstract and author keywords - adjx = within x words, regardless of order - * = truncation of word for alternate endings |
| --- | --- |
| 1. Offspring/  2. Daughters/  3. Sons/  4. (child* or adolescen* or daughter* or son or sons or offspring or teen*).ti,ab,id.  5. or/1-4  6. ((parent* or maternal* or paternal* or mother* or father* or famil*) adj4 (disorder* or illness* or ill or impairment or impaired or sick* or diagnos* or disease* or injur*)).ti,ab,id.  7. Family intervention/  8. exp Counseling/  9. Social support/  10. exp Communication/  11. ((disclos* or intervent* or support* or communicat* or counsel* or encounter* or telling or talking or conversation*) adj3 (adolescen* or famil* or child* or teen*)).ti,ab,id.  12. or/7-11  13. 5 and 6 and 12  14. remove duplicates from 13 | |

4. Cinahl

| Interface: Ebsco  Date of Search: 9 January 2018  Number of hits: 2,056 | Field labels   - MH+ = exploded Cinahl Heading - MH = non exploded Cinahl Heading - TI = title - AB = abstract - Nx = within x words, regardless of order - * = truncation of word for alternate endings |
| --- | --- |
| S1 (MH "Children of Impaired Parents")  S2 TI ( (parent* or maternal* or paternal* or mother* or father* or famil*) N3 (disorder* or illness* or ill or impairment or impaired or sick* or diagnos* or disease* or injur*)) ) OR AB ( (parent* or maternal* or paternal* or mother* or father* or famil*) N3 (disorder* or illness* or ill or impairment or impaired or sick* or diagnos* or disease* or injur*))  S3 TI ( (child* or adolescent* or teen* or offspring or daughter* or son or sons) ) OR AB ( (child* or adolescent* or teen* or offspring or daughter* or son or sons) )  S4 S2 AND S3  S5 S1 OR S4  S6 (MH "Professional-Family Relations")  S7 (MH "Counseling")  S8 (MH "Support, Psychosocial")  S9 (MH "Communication+")  S10 TI ( ((disclos* or intervent* or support* or communicat* or counsel* or encounter* or telling or talking or conversation*) N2 (adolescen* or famil* or child* or teen*)) ) OR AB ( ((disclos* or intervent* or support* or communicat* or counsel* or encounter* or telling or talking or conversation*) N2 (adolescen* or famil* or child* or teen*)) )  S11 S6 OR S7 OR S8 OR S9 OR S10  S12 S5 AND S11 | |

5. SveMed+

| Interface: https://svemedplus.kib.ki.se/  Date of Search: 9 January 2018  Number of hits: 91 | Field labels   - noexp = non exploded MeSH Heading |
| --- | --- |
| 1 noexp:”Child of Impaired Parents”  2 noexp:”Professional-Family Relations”  3 noexp:”Counseling”  4 noexp:”Social Support”  5 noexp:”Communication”  6 noexp:”Disclosure”  7 noexp:”Truth Disclosure”  8 #2 OR #3 OR #4 OR #5 OR #6 OR #7  9 #1 AND #8 | |
