# Supplementary material for: Informing children of their parent's illness: A systematic review of intervention programs with child outcomes in all health care settings globally from inception to 2019
Source: PLoS One. 2020 May 26;15(5):e0233696. doi: 10.1371/journal.pone.0233696 (PMC7250450; doi:10.1371/journal.pone.0233696)
Supplement: Attachment — Submitted filename: Response to Reviewers.docx [file pone.0233696.s005.docx]

To: Abraham Salinas-Miranda	7 April 2020
Academic Editor
PLOS ONE

Response to (Revision required [PONE-D-19-28601] - [EMID:1db6108ebab34e3a]

Dear Editor,

We were pleased to receive your letter conveying the responses from the reviewers. We greatly appreciate the reviewers’ comments that this review is important, new and well written.

Two reviewers inquire as to why we have not searched PubMed. This is a misunderstanding. PubMed was searched via the interface” Ovid MEDLINE(R) and Epub Ahead of Print, In-Process & Other Non-Indexed Citations and Daily.” The Library at Karolinska Institutet used this search interface as it offers more precise search options and accesses the same material as PubMed. It is important that the reader clearly understands that PubMed was searched. In accordance with the wording of the webpage of The National Library of Medicine we have chosen to change all mentions in our article of Medline to Medline/PubMed.

The reviewers correctly point out that “using the same wording in the objectives as in the conclusion would make it a clearer connection that both objectives” and “conclusions are aligned/the conclusion does not reflect the goals of the study, this should be corrected.” We are grateful for this observation and have rewritten the conclusions, page 30-31 in Manuscript with Track Changes.

Also correctly noted: “The identified concepts found in the systematic should be reflected in the abstract's result section”. Thank you for this comment and note our revised abstract page 3-4.

One reviewer comment: “Page no. 5 the law and policy protecting and supporting child development is varying from country to country so the review should be restricted to Sweden not globally.” Our main argument is that Transgenerational Transmission of Illness is a universal phenomenon. This is supported by the fact that the studies we found, conducted in different countries, are similar and have similar effects. Furthermore, there are global guidelines. In addition, some nations have national regulations on this matter. Sweden is taken as an example of such a nation. The title of the article “Informing Children of Their Parent’s Illness: A Systematic Review of Intervention Programs with Child Outcomes in All Health Care Settings Globally from Inception to 2019” refers to the global breath of the literature search we have undertaken. Unfortunately, there is not enough research in Sweden to warrant a systematic literature review. We hope to have made the passage on page 7 clearer by adding and adjusting line 117-118: In addition, some nations have national regulations guiding health care staff of adult patients to attend to needs of these patients’ children. For example, in Sweden…

The comment “Page no 11 PROSPERO registering no should be given along with the website address (already mentioned)” is difficult to understand. We have supplied the link to the registration on page 12, line 230-232. Is something more suggested for us to do?

The reviewer asks: Did the author try to get relevant data by contacting the authors of Non- English article? Thank you for the constructive comment. This was not found possible to do within this project. There was a massive amount of work to review the articles written in English found in literature searches. The authors prioritized publishing this paper in its current state.

We have included the following line in the article, page 14 line 277. “We did not contact authors of Non-English articles due to time and monetary constraints.”

You noted some minor occurrence of overlapping text with the following previous publication(s), which needs to be addressed:

We have checked the occurrences of overlapping text and they are all paraphrasing’s of articles we have duly referenced. We have chosen to use the wording of the article to restate the content as precisely as possible. For specifics, please see note 1, below in this letter.

We have now revised the Title page according to instruction.

Updated Funding Statement

The funder Capio (https://capio.com/en/) provided support in the form of salary for only the author Charlotte Oja but did not have any additional role in the study design, data collection and analysis, decision to publish, or preparation of the manuscript. The specific roles of the authors are articulated in the ‘author contributions’ section.

Updated Competing Interests Statement

Charlotte Oja is employed by Capio as a clinical Medical Doctor, Specialist of General Practice. This does not alter our adherence to PLOS ONE policies on sharing data and materials.

In our review of the manuscript we also discovered incorrect numbers concerning exclusions in Figure 1, which we now have corrected as well as minor errors in the descriptive text of Table 1.

We appreciate your positive response and constructive comments to this systematic literature review. In collaboration with the co-authors we have made the required changes to our review. We hope our improvements and clarifications are satisfactory and that the review soon can be available to readers.

Best regards,

Solvig Ekblad/ Charlotte Oja/ Anna Nager/ Tobias Edbom/ Jörgen Månson

# Note 1.

# http://bora.uib.no/handle/1956/15934 This link refers to our reference 23. We have described Gullbra’s findings in the wording of the article and made a reference to the article as 23. Gullbra F, Smith-Sivertsen T, Graungaard AH, Rortveit G, Hafting M. How can the general practitioner support adolescent children of ill or substance-abusing parents? A qualitative study among adolescents. Scand J Prim Health Care. 2016:1-8.

# https://www.tandfonline.com/doi/full/10.1080/02673843.2018.1548360 This links to our own publication Making life work for both themselves and their parents alone- how children aged 11–16 view themselves in relation to their chronically ill parents in a primary health care setting and is referenced to as part of the discussion: In previous research (60) it has been shown that illness can reverse the roles and make the child take over parental responsibilities and as 60. Oja C, Edbom T, Nager A, Månsson J, Ekblad S. Making life work for both themselves and their parents alone- how children aged 11–16 view themselves in relation to their chronically ill parents in a primary health care setting. International Journal of Adolescence and Youth. 2018:1-13.

# https://www.ncbi.nlm.nih.gov/pubmed?Cmd=ShowDetailView&Db=pubmed&TermToSearch=28505032 links to Do preventive interventions for children of mentally ill parents work? Results of a systematic review and meta-analysis. Thanhäuser M1 and is referenced by us as 19. Thanhauser M, Lemmer G, de Girolamo G, Christiansen H. Do preventive interventions for children of mentally ill parents work? Results of a systematic review and meta-analysis. Curr Opin Psychiatry. 2017;30(4):283-99. Corresponding to our article text: “A meta-analysis in 2017 (19) reported on 96 articles, including 50 independent samples from randomized controlled trials quantifying effects of preventive interventions. Random effect models resulted in small though significant Effect Sizes for programs enhancing mother-infant interaction as well as mothers’ and children’s behavior. Intervention programs for children/adolescents resulted in statistically significant small positive effects for global psychopathology as well as internalizing symptoms and this positive effect increased over time. Also externalized symptoms reached significance in the follow-up assessments. Interventions addressing parents and children jointly produced overall larger effects.”
